# Similar programmed death ligand 1 (PD-L1) expression profile in patients with mild COPD and lung cancer

**DOI:** 10.1038/s41598-022-26650-9

**Published:** 2022-12-27

**Authors:** F. Polverino, D. Mirra, C. X. Yang, R. Esposito, G. Spaziano, J. Rojas-Quintero, M. Sgambato, E. Piegari, A. Cozzolino, E. Cione, L. Gallelli, A. Capuozzo, C. Santoriello, L. Berrino, J. P. de- Torres, T. L. Hackett, M. Polverino, B. D’Agostino

**Affiliations:** 1 Pulmonary and Critical Care Medicine, Department of Medicine, Baylor College of Medicine, Houston, TX 77030 USA; 2 University of Campania “Luigi Vanvitelli”, Caserta, Italy; 3 University of British Columbia, Vancouver, Canada; 4 University of Calabria, Rende, Italy; 5 University of Catanzaro, Catanzaro, Italy; 6 Ospedale “M. Scarlato”, Scafati, Italy; 7 Ospedale Antonio Cardarelli, Naples, Italy; 8 Queen’s University, Hamilton, Canada

**Keywords:** Non-small-cell lung cancer, Chronic obstructive pulmonary disease

## Abstract

Programmed Death Ligand 1 (PD-L1) is crucial in regulating the immunological tolerance in non-small cell lung cancer (NSCLC). Alveolar macrophage (AM)-derived PD-L1 binds to its receptor, PD-1, on surveilling lymphocytes, leading to lymphocyte exhaustion. Increased PD-L1 expression is associated with cigarette smoke (CS)-exposure. However, the PD-L1 role in CS-associated lung diseases associated with NSCLC, such as chronic obstructive pulmonary disease (COPD), is still unclear. In two different cohorts of ever smokers with COPD or NSCLC, and ever and never smoker controls, we evaluated PD-L1 expression: (1) via cutting-edge digital spatial proteomic and transcriptomic profiling (Geomx) of formalin-fixed paraffin-embedded (FFPE) lung tissue sections (n = 19); and (2) via triple immunofluorescence staining of bronchoalveolar lavage (BAL) AMs (n = 83). PD-L1 mRNA expression was also quantified in BAL AMs exposed to CS extract. PD-L1 expression was increased in the bronchiolar wall, parenchyma, and vascular wall from mild-moderate (GOLD 1–2) COPD patients compared to severe-very severe (GOLD 3–4) COPD patients and controls. Within all the COPD patients, PD-L1 protein expression was associated with upregulation of genes involved in tumor progression and downregulation of oncosuppressive genes, and strongly directly correlated with the FEV_1_% predicted, indicating higher PD-L1 expression in the milder vs. more severe COPD stages. In bronchioles, PD-L1 levels were strongly directly correlated with the number of functionally active AMs. In BAL, we confirmed that AMs from patients with both GOLD 1–2 COPD and NSCLC had the highest and similar, PD-L1 expression levels versus all the other groups, independently from active cigarette smoking. Intriguingly, AMs from patients with more severe COPD had reduced AM PD-L1 expression compared to patients with mild COPD. Acute CS extract stimulation increased PD-L1 mRNA expression only in never-and not in ever-smoker AMs. Lungs from patients with mild COPD and NSCLC are characterized by a similar strong PD-L1 expression signature in bronchioles and functionally active AMs compared to patients with severe COPD and controls. Active smoking does not affect PD-L1 levels. These observations represent a new resource in understanding the innate immune mechanisms underlying the link between COPD and lung cancer onset and progression and pave the way to future studies focused on the mechanisms by which CS promotes tumorigenesis and COPD.

## Introduction

One of the core functions of the immune system is the discrimination between self and non-self, necessary to protect our body against external noxious antigens^1^. This mechanism is regulated by a subtle balance between immune regulatory and effector cells that, when blunted, leads to immune responses against autologous antigens^2^. In this context, an important role is played by immune checkpoints, which participate to the immune tolerance by preventing reactions against “self,” or autoimmunity^3^. Specifically, programmed death 1 (PD-1) and its ligands PD-L1 and PD-L2 promote self-tolerance by suppressing branches of the T cell compartment during immune responses^3^. Recent studies have suggested that alveolar macrophage (AMs) expression of PD-L1 contributes to the regulation of the immunological tolerance in non-small cell lung cancer (NSCLC)^4,5^. PD-L1 binds to its receptor, PD-1, on surveilling lymphocytes, leading to lymphocyte exhaustion, a state of impaired function^6^.

Chronic Obstructive Pulmonary Disease (COPD) is characterized by progressive airflow limitation associated with an abnormal pulmonary and systemic immune response to particles or gases such as cigarette smoke (CS)^7^. COPD is considered a risk factor for NSCLC^8^, and both diseases may have a common etiology^9^. Although many studies highlight the epidemiological links between COPD and lung cancer, pointing at CS as a common risk factor for both, the molecular bases of this association are less well defined^10^. If, on the one hand, CS elicits an inflammatory response in the lung of all smokers, leading to the release of immunogenic antigens such as elastin^11^, not all smokers react against these autologous antigens. Among those who do react, its intensity varies, thus accounting for the wide range of disease manifestations, such as COPD (emphysema and chronic bronchitis) or lung cancer^12–15^. In those who develop COPD, the pulmonary inflammation often persists for years after smoking cessation, indicating blunted immune tolerance mechanisms in these patients^13^. Interestingly, among patients with established clinical COPD, lung cancer occurs more frequently in the ones with milder COPD^15^. Thus, recent studies have pointed at a crucial role for innate and adaptive immune responses as the tip of the balance regulating the development of COPD or NSCLC phenotypes in response to CS^16,17^. AMs are crucial mediators of lung immune responses to CS within the innate immune system, for example, by orchestrating T cell functions ^4,5^. Therefore, in the current study we evaluated, via cutting-edge spatial profiling and immunostaining, PD-L1 expression in AMs from patients with COPD, NSCLC, and ever- or never- smoker controls in vivo and tested the effects of CS on AMs from each patients’ group in vitro.

## Materials and methods

### Cohort #1: Thoracic surgery cohort- digital spatial profiling

#### Study population

We collected lung specimens from 19 subjects undergoing lung volume reduction surgery or transplant to treat severe emphysema, or lung wedge resection for a solitary peripheral nodule (the lung tissue studied was at least 10 cm away from the nodule). All the subjects defined as ever-smokers had a smoking history of at least ten packs/years and quit smoking at least one year prior to the study. The study subjects were classified as follows: (1) never-smoker controls (“NS”, n = 4); smoker controls (“smokers”, n = 5); smokers with Global Initiative for Obstructive Lung Diseases (GOLD) stage 1–2 COPD (n = 7); smokers with GOLD stage 3–4 COPD (n = 3). The diagnosis of COPD was performed according to the GOLD international recommendations^18^. All the subjects underwent spirometry according to international guidelines. The exclusion criteria were evidence of respiratory tract infection at the time of lung tissue sampling, presence of concomitant chronic lung disease or metastatic cancer, autoimmune disease, immunosuppressive therapy, or chemotherapy (Table 1).

**Table 1:** Demographic characteristics thoracic surgery cohort (n = 19).

	NS	Smokers	GOLD 1–2	GOLD 3–4	*P*-value
			COPD	COPD	
**Total partecipants (N)**	4	5	3	7	
Age	63 (8)	76 (6)	70 (7)	65 (5)	NS
Gender (M/F)	2/2	3/2	2/1	5/2	NS
Smoking history (pack years)	0	30 (15)	30 (10)	44 (33)	< 0.001*
Smoking habit (current/former smoker)	0/4	1/4	0/3	0/7	NS
FEV1 (% predicted)	91% (16)	94% (19)	67 (16)	29% (18)	**< 0.001**^#^
FEV/FVC	88 (5)	78 (4)	71 (8)	38 (21)	**< 0.001**^#^
**Comorbidities**	7	3	1	8	
Hypertension (%)	3 (75%)	2 (50%)	0	3 (43%)	NS
Other cardiovascluar diseases (%)	2 (50%)	1 (25%)	1 (33%)	4 (57%)	NS
Diabetes Mellitus (%)	2 (50%)	0	0	1 (14%)	NS
**Medications**	1		2	16	
Inhaled corticosteroids (N, %)	0	0	1 (33%)	5 (71%)	**< 0.05**^+^
LABA/SABA/LAMA (N, %)	0	0	1 (33%)	7 (100%)	**< 0.05**^+^
Oral corticosteroids (N, %)	0	0	0	3 (43%)	**< 0.05**^+^
Ca^+^-antagonists (N, %)	1 (25%)	0	0	1 (14%)	NS

Data are mean ± SD, unless specified.

NS = Never-smokers; COPD: chronic obstructive pulmonary disease; GOLD: global initiative for chronic lung diseases.

LABA: Long-acting beta-agonists; SABA: short-acting beta-agonists; LAMA: long-acting muscarinic agents; ACEi: ACE-inhibitors; ARB: angiotensin receptor blockers.

*p value = smokers, GOLD 1–2 COPD, and GOLD 3–4 COPD versus NS.

^#^*p* value = GOLD 3–4 COPD versus all the groups, and GOLD 1–2 versus NS, by definition. GOLD 1–2 versus smokers = 0.06.

^+^GOLD 3–4 COPD versus controls.

Significant values are in bold.

#### Nanostring GeoMx digital spatial proteomic and transcriptomic profiling

**Figure 1:**
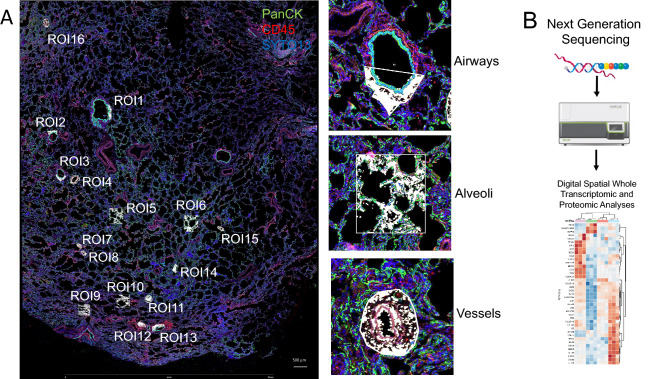
NanoString GeoMx spatial protein and transcriptomic profiling: (**A**) Image of a lung tissue section from a representative COPD subject stained with pan-cytokeratin (green), CD45 (red) and SYTO13 (blue). The regions of interest (ROIs) sampled for spatial protein and RNA analysis are highlighted in the white boxes. (**B**) The workflow of protein and RNA data analyses is shown.

Formalin-fixed and paraffin-embedded (FFPE) lung tissue sections from each subject were obtained. The sections were deparaffinized, and antigen retrieval was performed using 1 × Citrate Buffer pH 6.0. Up to sixteen tissue regions of interest (ROIs) encompassing a range of morphological tissue features were selected for genome-wide transcriptomic and 40-plex antibody profiling based on enrichment for SYTO13, pan-cytokeratin, or CD45 expression (Fig. 1A). Three main types of ROIs were selected: parenchyma, bronchiole, and vessel tissues. The selection of the ROI was done taking into account histological delimitations; airways were selected in Pan-CK+ cells down to the basal lamina, and endothelial cells were chosen from Pan-CK- cells surrounded by elastic layers or vasa vasorum. The DSP machine is capable of a zoom of up to 50 microns, which allows ample magnification of the tissue and the neat selection of ROIs. The machine allows us to select areas as small as 10 cells, and as big as 3000 cells (maximum 660 microns in diameter). Once the ROI is chosen, the GeoMx uses its mirrors to locate the section with surgical precision. The samples were then incubated with the GeoMx Transcriptome Whole Transcriptome Atlas Panel for RNA studies, and 41 oligo-labeled primary antibodies for protein studies (See Table 2) composed of: a Human Immune Cell Profiling Core, a Human Immune Activation Status Panel, a Human Immune Cell Typing Panel, a COVID-19 Immune Monitoring Panel, and custom-labeled antibodies against Syndecan-1, CD10, CD21 (all from Abcam, Cambridge, MA). The slides were then loaded onto the DSP, and the maximum number of ROIs were selected per slide. A total of 192 ROIs were analyzed for RNA and protein expression. All the indexing oligonucleotides were collected into a 96 well plate and were then hybridized to fluorescent barcodes using GeoMx Hyb Codes. After hybridization, samples were processed using the nCounter (Nanostring®) for protein analyses, and NextSeq 500 System (Illumina®) according to the manufacturer’s instructions, and whole transcriptomic and proteomic data were generated for each ROI for each of the study subjects (Fig. 1B). In order to validate the findings, double immunofluorescence staining for PD-L1 (E1L3N® XP® Rabbit mAb #13684, Cell Signaling, Danvers, MA) and (CD68E-11, mAb #17832, Santa Cruz Biotecnologies, Dallas TX) was performed in the same patient population. PD-L1 expression in AMs in alveoli, bronchi, and vessels was quantified with Metamorph Software (Molecular Devices, San Jose, CA) according to a previously published protocol^19^.

**Table 2:** Nanostring GeoMx protein targets.

Protein	Code class	Protein group
S6	Control	Housekeepers; all targets
Rb IgG	Negative	Background; all targets
Ki-67	Endogenous	Proliferation; all targets
CD45	Endogenous	Total Immune; all targets
PD-1	Endogenous	T cells; checkpoint; T cell activation; all targets
CD68	Endogenous	M2 Macrophage; myeloid; macrophage; all targets
GZMB	Endogenous	T cell activation; cytotoxicity; all targets
Ms IgG1	Negative	Background; all targets
GAPDH	Control	Housekeepers; all targets
Histone H3	Control	Housekeepers; all targets
CTLA4	Endogenous	T cells; checkpoint; T cell activation; Th cells; all targets
PD-L1	Endogenous	Checkpoint; myeloid activation; all targets
Fibronectin	Endogenous	Stroma; fibroblasts; all targets
CD20	Endogenous	B cells; all targets
CD4	Endogenous	T cells; myeloid; Th cells; all targets
CD8	Endogenous	T cells; CD8 T cells; all targets
Ms IgG2a	Negative	Background; all targets
HLA-DR	Endogenous	Antigen presentation; MHC2; all targets
CD3	Endogenous	T cells; all targets
PanCk	Endogenous	Tumor; epithelial; all targets
Beta-2-microglobulin	Endogenous	Tumor; antigen presentation; all targets
CD11c	Endogenous	DC; myeloid; all targets
SMA	Endogenous	Stroma; all targets
CD56	Endogenous	NK cells; all targets
Cathepsin L/V/K/H	Endogenous	Protease; all targets
TMPRSS2	Endogenous	Protease; all targets
DDX5	Endogenous	Immune response; all targets
ACE2	Endogenous	Viral receptor; all targets
CD27	Endogenous	T cells; T cell activation; all targets
CD80	Endogenous	Myeloid; myeloid activation; all targets
CD40	Endogenous	Myeloid; myeloid activation; all targets
CD44	Endogenous	T cell activation; all targets
CD25	Endogenous	T cells; T cell activation; Tregs; all targets
PD-L2	Endogenous	Checkpoint; all targets
CD127	Endogenous	T cells; naive and memory; all targets
ICOS	Endogenous	T cell activation; all targets
CD14	Endogenous	Myeloid; monocyte; all targets
CD45RO	Endogenous	T cells; memory; all targets
FOXP3	Endogenous	T cells; Th cells; tregs; all targets
CD34	Endogenous	Hematopoietic; all targets
FAP-alpha	Endogenous	Stroma; fibroblasts; all targets
CD163	Endogenous	M2 Macrophage; myeloid; macrophage; all targets
CD66b	Endogenous	Myeloid; neutrophil; all targets
CD21	Endogenous	Custom target; all targets
Syndecan-1	Endogenous	Custom target; all targets
CD10	Endogenous	Custom target; all targets

### Cohort #2: Bronchoscopy cohort: immunofluorescence and in vitro studies

#### Study population

To understand the specific effects of NSCLC on PD-L1 expression, we performed a prospective study on 190 age- and sex-matched subjects, aged 18 years or older, referred to the Pulmonary and Critical Care Medicine Department of “Mauro Scarlato” Hospital in Scafati, Italy, with a suspected diagnosis of pulmonary neoplasia undergoing routine bronchoscopy and bronchoalveolar lavage (BAL). This study is a part of the clinical trial recorded in clinicaltrials.gov (NCT04654104) and approved by the local Ethics Committee “Calabria Centro” and “ASL Salerno”. This work was conducted in compliance with the Institutional Review Board/Human Subjects Research Committee requirements and the Declaration of Helsinki and the Guidelines for Good Clinical Practice criteria. Before the beginning of the study, all the enrolled patients or legal guardians signed the informed consent. Patients’ demographics and clinical and social history were obtained at enrollment. Subjects with active pulmonary infections, autoimmune diseases, extrapulmonary neoplasia, or others airflow obstruction, e.g., asthma or bronchiectasis, or did not sign the informed consent at the time of enrolment were excluded. The remaining 83 subjects were then classified into five groups, according to their clinical and pathological (bronchoscopy-guided) diagnosis (See Table 3): (1) Healthy never smokers (“NS”. n = 16); (2) smokers without COPD or NSCLC (“smokers” n = 17); (3) never-smokers with NSCLC (“NS + cancer; n = 12); (4) ever smokers with NSCLC (“smokers + cancer”, n = 22); (5) smokers with GOLD 1–2 COPD (“GOLD 1–2 COPD”, n = 9) and (6) smokers with GOLD 3–4 COPD (GOLD 3–4 COPD”, n = 7).

**Table 3:** Demographic characteristics bronchoscopy cohort (n = 83).

	NS	Smokers	NS + Cancer	Smokers + Cancer	GOLD 1–2	GOLD 3–4	*P*-value
					COPD	COPD	
Total participants (N)	16	17	12	22	9	7	
Mean age (years)	66 (17)	61 (9)	71 (9)	69 (7)	67 ± (9)	69 (7)	NS
Gender (M/F)	4/12	14/3	7/5	17/5	4/5	6/1	NS
Smoking history (pack/years)	0	30.8 (17)	0	30 (16)	25 (7)	19 (16)	**< 0.001**^#^
Smoking habit (current/former smoker)	0/16	6/11	0	15/7	4/5	2/5	**< 0.05**^**+**^
FEV1.0 (% predicted)	97 (5.34)	86 (4.52)	92 (7.9)	90 (10.24)	68 (8.33)	43 (4.6)	< 0.05*
FEV/FVC ratio	75.1 (2.3)	77.3 (3.4)	78.9 (4.1)	77.3 (3.4)	58.2 (5.2)	39.1 (6.7)	< 0.05*
**Comorbidities**	15	15	9	19	7	7	NS
Hypertension N (%)	10 (62%)	6 (35%)	6 (50%)	6 (27%)	3 (33%)	3 (43%)	NS
Other cardiovascluar diseases N (%)	4 (25%)	5 (29%)	2 (17%)	9 (41%)	2 (22%)	4 (57%)	NS
Diabetes mellitus N (%)	3 (19%)	4 (23%)	1 (8%)	4 (33%)	2 (22%)	0	NS
**Medications**	13	15	8	17	8	7	NS
Inhaled corticosteroids N (%)	1 (6%)	0	0	0	2 (22%)	2 (29%)	**< 0.001**^+^
LABA/SABA/LAMA N (%)	2 (12%)	0	0	2 (9%)	7 (78%)	5 (71%)	**< 0.001**^#^
Oral corticosteroids N (%)	3 (19%)	1 (6%)	6 (50%)	0	0	1 (14%)	**< 0.05**^#^
ACEi/ARB N (%)	4 (25%)	6 (35%)	7 (58%)	3 (14%)	1 (11%)	2 (29%)	**< 0.000**1^#^
Ca2 + Antagonists N (%)	1 (6.25%)	1 (5.88%)	1 (8.33%)	1 (4.54%)	0	1 (14.28%)	**< 0.05**^#^
Diuretics N (%)	3 (19%)	4 (25%)	5 (42%)	3 (14%)	1 (11%)	2 (29%)	**< 0.001**^#^

Data are mean ± SD, unless specified.

NS = Never-smokers; COPD: chronic obstructive pulmonary disease; GOLD: global initiative for chronic lung diseases.

LABA: Long-acting beta-agonists; SABA: short-acting beta-agonists; LAMA: long-acting muscarinic agents; ACEi: ACE-inhibitors; ARB: angiotensin receptor blockers.

*p value = GOLD 3–4 vs all the other groups, GOLD 1–2 versus NS, Smokers, Smokers + cancer, GOLD 3–4, by definition.

^+^*p* value = GOLD 3–4 versus all the other groups, GOLD 1–2 versus NS, Smokers, GOLD 3–4.

^#^*p* value = GOLD 3–4 versus all the other groups, GOLD 1–2 versus all the other groups.

Significant values are in bold.

### Bronchoscopy and bronchoalveolar lavage

All subjects underwent bronchoscopy for clinical indications, and BAL was obtained with a flexible bronchoscope according to internationally accepted guidelines^20^. The procedure involved premedication (20 mg codeine *per os*) and local anesthesia of the larynx and lower airways (0.5% tetracaine in the oropharynx, 8 cc 0.5% tetracaine in lower airways). Transcutaneous oxygen saturation was monitored continuously by an oximeter with a finger probe. BAL was performed in the right middle lobe with a total volume of 200 ml of sterile isotonic saline solution (37 °C). BAL fraction I, returned after instilling 50 ml of saline, and BAL fraction II, returned after instilling 3 × 50 ml of saline, was collected in a siliconized specimen trap and immediately kept on ice. BAL fluid fractions were filtered through nylon gauze and centrifuged (10 min at 400 g at 4 °C). The cell pellet was washed twice, counted, and resuspended in PBS. Cells were counted in a Bürker chamber. Cell yield was determined by total cell number per fraction/total recovered volume per fraction. Cell viability was determined by Trypan blue exclusion. Smears for cell differentiation were prepared by cytocentrifugation (Shandon, Runcorn, UK). Cell differentiation was performed by microscopy on the cytospin slide after staining with QUICK-DIFF KIT; at least 100 cells were counted.

### PD-L1 Immunofluorescence on BAL alveolar macrophages (AMs)

PD-L1 expression was assessed in AMs obtained from BAL. After washing cytocentrifuge preparation slides in PBS, non-specific binding sites were blocked by treatment with donkey serum for 30 min. The slides were incubated overnight at 4 °C with a primary murine monoclonal antibody directed against CD68 (as activated AMs marker, Thermo Fisher Scientific, dilution 0.2 mg/mL). After several rinses in phosphate-buffered saline, the slides were exposed to FITC-conjugated goat anti-mouse (Abcam Donkey Anti-Mouse IgG, FITC) and incubated at 37° for 1 h. Then, the slides were rinsed in phosphate-buffered saline and incubated overnight at 4 °C with a primary rabbit monoclonal antibody directed against PD-L1 (Thermo Fisher Scientific, dilution 1 mg/mL). After several rinses in PBS, the slides were exposed to TRITC-conjugated goat anti-rabbit (Abcam Donkey Anti-Rabbit IgG, TRITC). The immunostainings were assessed with a fluorescence microscope (Nikon Eclipse E-600) using a 60X objective. To overcome a sampling bias, the quantification of PD-L1+ AMs was performed on three consecutive sections. The results were expressed as an average ± SEM of three independent counts.

### In vitro studies

To test the effect of active smoking on PD-L1 macrophage expression, we performed the following in vitro studies with THP-1 macrophage cell line and AMs harvested from 5 NS, 4 smokers, 5 smokers + NSCLC, and 4 COPD patients as outlined below.

#### Preparation of CS extract

Cigarette smoke extract (CSE) was prepared using Red Marlboro cigarettes (Phillip Morris; Cracow, Poland) containing 8 mg of tar, 0.6 mg of nicotine, and 9 mg of carbon monoxide per cigarette as previously described^19^. In brief, ten cigarettes without filter were bubbled through a total of 250 ml of serum-free RPMI with a modified vacuum pump apparatus. The resulting suspension was adjusted to pH 7.4 and then filtered through a 0.20 µm pore filter to remove bacteria and large particles^19^.

#### PD-L1 mRNA expression in THP-1 cells and AMs

A pilot in vitro model of macrophages from acute monocytic leukemia (THP-1)^21^ was used to assess the exact dose (whether 10%, 20% or 40%) of CSE was able to trigger PD-L1 mRNA expression at 24 h, 48 h or 72 h. The THP-1 cell line was obtained from ATCC and maintained at 2 × 10^5^ cells/ml in RPMI-1640 medium supplemented with 10% FBS and 2 mM L-glutamine. THP-1 cells were differentiated using 100 ng/ml phorbol 12-myristate 13-acetate (PMA, Sigma-Aldrich) for two days. Afterward, the cells were re-fed with fresh medium without PMA for 1 day to allow cell recovery. Differentiated THP-1 were then exposed to 10%, 20% or 40% CSE medium for 24 h, 48 h or 72 h. Then, in a subset of patients (5 NS, 4 smokers, 5 smokers + NSCLC, and 4 COPD (GOLD 1–4) patients, we assessed CSE-induced PD-L1 mRNA levels in BAL-derived AMs. BAL cell pellets were washed three times with PBS and suspended with RPMI-1640 containing 10% FBS, 2 mM L-glutamine, 200 U/ml penicillin, and 200 mg/ml streptomycin. The cell suspension was added at 0.5 × 10^6^ cells/well to a 75-plastic tissue culture flask and incubated at 37 °C in a 5% CO_2_ humidified milieu for 2 h to permit the adherence of AMs. Then the non-adherent cells were removed by three washes with PBS. The purity of adherent AMs was identified as greater than 95% by morphology. Then the AM were exposed to 10% CSE for 24 h, previously set by the pilot in vitro THP-1 cultures. Total RNA was extracted from AMs and THP-1 cells by Trizol according to the manufacturer's instructions. Both cDNA synthesis and PCR were performed simultaneously using the SuperScript III Platinum SYBR Green One-Step qRT-PCR Kit (Invitrogen) using the CFX96 real-time system (Bio-Rad Laboratories). The transcript levels of PD-L1 were detected and the housekeeping gene encoding GAPDH was used as an internal control for mRNA expression studies. Relative expression was calculated using the comparative cycle threshold (Ct) method (2 − ΔΔCt).

### Statistical analysis

For the digital spatial profiling analyses, proteins with reliable expression in at least ten samples (expression level > the average expression of the negative control probes) were included in the downstream analyses. Differential protein expression analysis was performed to identify tissue markers (parenchyma, bronchiole, or vessel tissues) and to compare the disease conditions using the Two-stage step-up method of Benjamini, Krieger, and Yekutieli (2006). A false discovery rate (FDR) of < 30% was used as the significance threshold. GraphPad Prism 9 InStat (GraphPad Software Inc., San Diego, CA) was used for statistical computations. Significance among multiple comparisons was determined by the one-way ANOVA corrected with Bonferroni's posttest^22^. Data that were not normally distributed are presented as median and interquartile range (IQR), and pairwise testing was performed using the Mann–Whitney U test. Normally distributed data are presented as mean, and SEM and pairwise testing were performed using the Student’s t-test. Correlations were calculated using the Spearman correlation test. A value of *P* < 0.05 was considered significant.

Linear model was used to identify genes that were associated with the up-regulated proteins in GOLD 1–2 compared to GOLD 3–4 in each tissue type and genes that were associated with PD-L1 protein expression in all the samples. Cytoscape (version 3.9.1) was used to create the Gene-Protein association networks. R package “fgsea” was used to identify C7 (immunologic signature gene sets) pathways that were enriched in PD-L1 protein associated genes.

## Results

The clinical characteristics of the subjects in both cohorts are reported in Tables 1 and 3.

### Digital spatial transcriptomic and proteomic profiling of lung tissue

#### Spatial proteomics lung tissue profiling

Figure 1 shows an example of a panorama FFPE section stained with pan-cytokeratin (epithelial marker), CD45 (leukocyte marker), and SYTO13 (DNA/nuclear marker) to enable sampling of upto 16 ROIs in the parenchymal, bronchioles, and vessels per each subject. From the 41 proteins assessed, 36 could be reliably measured compared to the endogenous and negative controls. For validation, we confirmed that each tissue type (parenchyma, bronchioles, vessel) expressed tissue-specific protein signatures across the multiple ROIs sampled. For example, vessels expressed higher levels of alpha-smooth muscle actin, whereas alveoli and bronchi expressed higher levels of pan-cytokeratin (a marker of lung epithelial cells).

**Figure 2:**
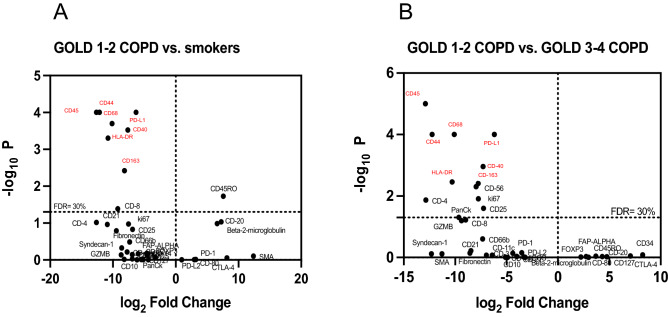
Top protein targets identified with spatial profiling: Volcano Plot showing the proteins above a false discovery rate of 30% in peripheral lungs (across all the regions of interest) of smokers without COPD versus GOLD 1–2 COPD (**A**) and GOLD 1–2 COPD versus GOLD 3–4 COPD (**B**). The x-axis is the log2 fold change, whereas the y-axis is the log10 of the p-value. In red, the most significantly up-regulated proteins in GOLD 1–2 COPD patients versus smokers (**A**) and GOLD 3–4 COPD (**B**).

As exploratory analyses, we ranked the main differences in protein expression between the four groups of subjects across all the ROIs. The patient group that had the highest number of proteins different vs. the other groups was the GOLD 1–2 COPD group. Of all the proteins above 30% FDR, PD-L1 (immune checkpoint marker), CD68 (a marker of functionally active macrophages (29), CD163 (a marker of functionally inactive macrophages (29), CD40 and CD44 (co-stimulatory B and T cell molecules expressed during viral infections), CD45 (pan-leukocyte marker), and Human Leukocyte Antigen–DR isotype (HLA-DR, ligand of T cell receptor) were significantly higher in the GOLD 1–2 COPD group vs. smokers (Fig. 2A) and GOLD 3–4 COPD group (Fig. 2B) across all the ROIs.

**Figure 3:**
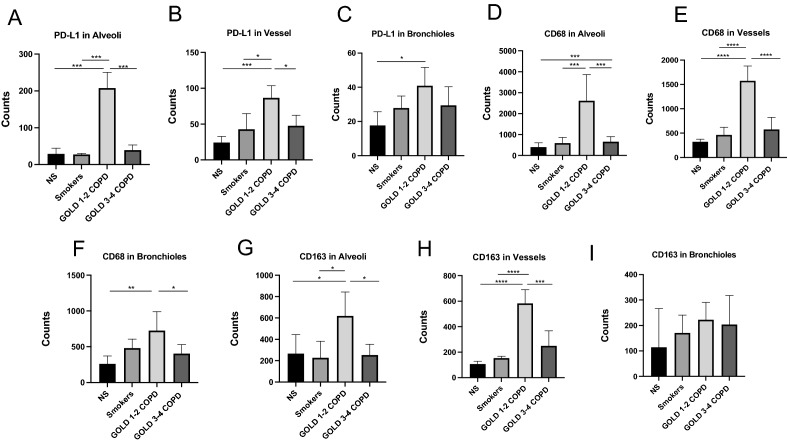
PD-L1 and CD68 are upregulated in peripheral lungs from GOLD 1–2 COPD patients. Digital spatial PD-L1 protein expression in alveoli (**A**), vessel (**B**) and bronchioles (**C**) from never smokers (NS), smokers, and GOLD 1–2 and GOLD 3–4 COPD patients. Results were shown as means ± SEM. The statistical tests used in these analyses was one-way analysis of variance followed by Bonferroni's posttest. *P < 0.05, ***P < 0.001. Digital spatial CD-68 protein expression in alveoli (**D**), vessel (**E**) and bronchioles (**F**) from never smokers (NS), smokers, and GOLD 1–2 and GOLD 3–4 COPD patients. Results were shown as means ± SEM. The statistical tests used in these analyses was one-way analysis of variance followed by Bonferroni's posttest. *P < 0.05, **P < 0.01, ***P < 0.001, ****P < 0.0001. Digital spatial CD-163 protein expression in alveoli (**G**), vessel (**H**) and bronchioles **(I**) from never smokers (NS), smokers, and GOLD 1–2 and GOLD 3–4 COPD patients. Results were shown as means ± SEM. The statistical tests used in these analyses was one-way analysis of variance followed by Bonferroni's posttest. *P < 0.05, ***P < 0.001, ****P < 0.0001.

Once the top candidate proteins were identified, we analyzed each top-protein expression in each type of ROI (alveoli, bronchioles, and vessels) separately. GOLD 1–2 COPD patients had greater expression of PD-L1 in alveoli and vessels than patients with GOLD 3–4 COPD patients, NS, and smokers (Fig. 3A,B). PD-L1 expression was also increased in the bronchiolar wall from GOLD 1–2 COPD patients compared to the other groups, although it reached the statistical significance only versus NS due to the sample size (Fig. 3C). CD68 expression was greater in alveoli and vessels from patients with GOLD 1–2 COPD than patients with GOLD 3–4 COPD, NS, and smokers (Fig. 3D,E). CD68 was also significantly increased in the bronchioles from GOLD 1–2 COPD patients vs. patients with GOLD 3–4 COPD and NS (Fig. 3F). CD163 followed the same pattern, with greater CD163 expression in alveoli and vessels from patients with COPD and NSCLC than patients with GOLD 1–2 COPD, NS, and smokers (Fig. 3G,H). Surprisingly, a different pattern was observed in bronchioles where, unlike CD68, CD163 expression was similar between the four groups of patients (Fig. 3I).

**Figure 4:**
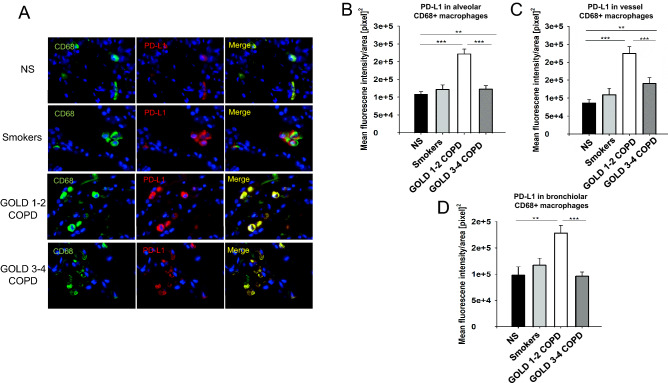
PD-L1 is overexpressed in peripheral lung AMs from GOLD 1–2 COPD patients: Immunofluorescence analysis of PD-L1 expression in CD68^+^ AMs in peripheral lungs from Cohort 1. (**A**) representative immunofluorescence pictures showing CD68+ (green, as a marker of macrophages) and PD-L1+ (red) cells. Nuclear DNA was labeled with DAPI (blue). (**B**) Mean fluorescence intensity of PD-L1 in alveoli from never-smokers (NS), smokers without COPD, GOLD 1–2 and 3–4 COPD patients. (**C**) mean fluorescence intensity of PD-L1 in vessels from never-smokers (NS), smokers without COPD, GOLD 1–2 and 3–4 COPD patients. (**D**) Mean fluorescence intensity of PD-L1 in bronchioles from never-smokers (NS), smokers without COPD, GOLD 1–2 and 3–4 COPD patients. *P < 0.05, **P < 0.01, ***P < 0.001.

Using double immunofluorescence staining, we confirmed that AMs in alveoli, vessels, and bronchioles from GOLD 1–2 COPD patients had higher expression of PD-L1 compared to the other patients’ groups, independently on the number of total AMs found in the areas (Fig. 4A–D).

**Figure 5:**
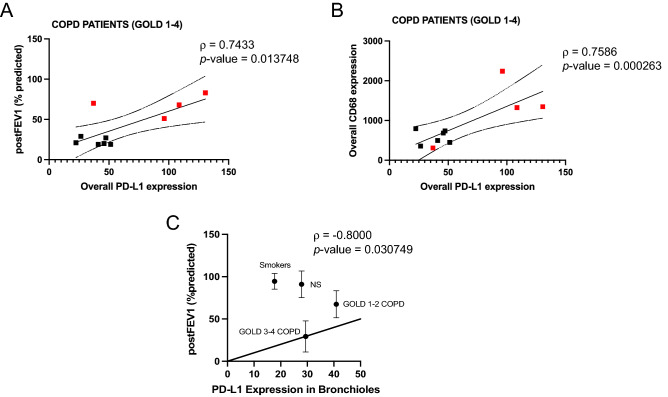
PD-L1 expression directly correlates with lung function and with CD68 expression: Correlation between total spatial PD-L1 expression levels and post-FEV1 (% predicted) (**A**) and spatial PD-L1 and CD68 expression levels (**B**) across all the COPD patients. Results were shown as means ± SEM. Red squares indicate GOLD 1–2 COPD patients. (**C**) the correlation between spatial PD-L1 expression and post-FEV1 (% predicted) in bronchioles across all the subjects’ groups is shown. The statistical tests used in these analyses was Spearman correlation test considering 95% confidence interval.

Within the COPD patients, the overall (across all the ROIs) PD-L1 protein expression was strongly directly correlated with the Forced Expiratory Volume in the 1 s (FEV1% predicted, which represents the proportion of a person's air that they can expire in the first second of forced expiration), indicating higher PD-L1 expression in the milder stages of the disease (Fig. 5A). Furthermore, PD-L1 expression was strongly directly correlated with the number of CD68^+^ macrophages (Fig. 5B). These correlations were not maintained were examining all the subjects (COPD and controls) together (Supplemental Fig. S1A,B). When we correlated PD-L1 expression in each type of ROI (bronchioles, vessels, and alveoli) with lung function (FEV1% predicted) across all the subjects, we found the strongest association in the bronchioles (Fig. 5C), unlike vessels and alveoli (Supplemental Fig. S2A,B, respectively). This strong direct correlation between PD-L1 and FEV1% predicted was present only in the COPD groups whereas it was lost when considering the NS and smokers groups separately (Supplemental Fig. S2C,D).

#### Network of genes and proteins within lung alveolar, bronchioles, and vessel tissues

**Figure 6:**
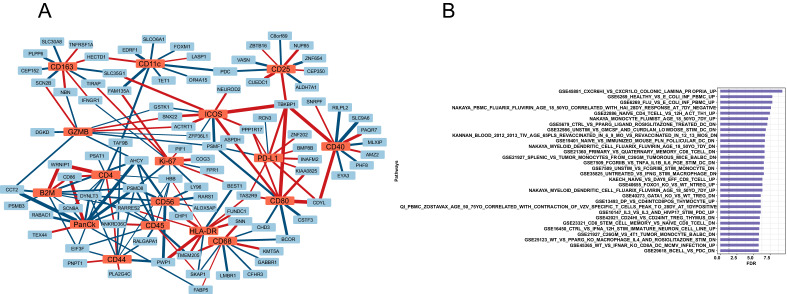
Network of associations between top protein and genes and pathway analysis: (**A**) Alveoli-specific RNA–protein association network (restricted to top 10 RNAs per protein, ranked by *p*-value). Red nodes = Up-regulated proteins in GOLD 1–2 compared to GOLD 3–4; Blue nodes = Associated RNAs; Red edges = positive association between the RNA and the protein; Blue edges = negative association between the RNA and the protein; Edge widths are proportional to the association effect sizes. (**B**) the top 30 (ranked by *p*-value) C7 (immunologic signature gene sets) pathways enriched in PD-L1 associated genes. The vertical dotted line indicates a FDR = 0.05.

The network plots in Fig. 6A and Supplemental Fig. S3A and B demonstrate all the tissue-specific top RNA–protein relationships. The red nodes indicate up-regulated proteins in GOLD 1–2 compared to GOLD 3–4, whereas the blue nodes indicate the associated genes. In alveoli (Fig. 6A), CD68 protein was positively and strongly associated, among others, with genes involved in cancer development such as *KTM5A*^23^ or *TMEM205*^24^. PD-L1 protein was spatially clustered with CD80 and ICOS proteins, markers of activated T cells, and both were strongly associated with genes involved in cancer progression, immunosuppression, and chemoresistance such as *CDYL*^25^ and *TBKBP1*^26^. Also, HLA-DR, which was upregulated in GOLD 1–2, was strongly associated with *TMEM205* and with *PWP1*, another gene upregulated in malignant lung cancer^27^.

In vessels (Supplemental Fig. S3A), PD-L1 was associated with downregulated expression of genes involved in T cell activation and polarization such as *CCT2*^28^ *and ITGBL1*^29^. PD-L1, CD-68, and HLA-DR were associated with downregulation of uncompressing gene such as *MTA3*^30^, and genes associated with lymphocyte proliferation such as *FAM221B*^31^. In bronchioles (Supplemental Fig. S3B), CD68 was associated with downregulation of oncosuppressing genes such as *TET1*^32^, *MIGA2*^33^, and *MTO1*^34^.

The Pathway analysis in Fig. 6B identified that the PD-L1 protein-associated genes were involved several pathways including IFN-gamma macrophage responses, switching between M1 and M2 macrophages, and T cell activation. When we narrowed our analysis to the macrophage-related pathways enriched in PD-L1 associated genes (Supplemental Fig. S3C), we confirmed that the top pathways found in GOLD 1–2 COPD patients were associated with PPAR-γ-induced macrophage migration and activation^35^.

### Immunofluorescence on BAL samples

#### PD-L1 expression on AMs

**Figure 7:**
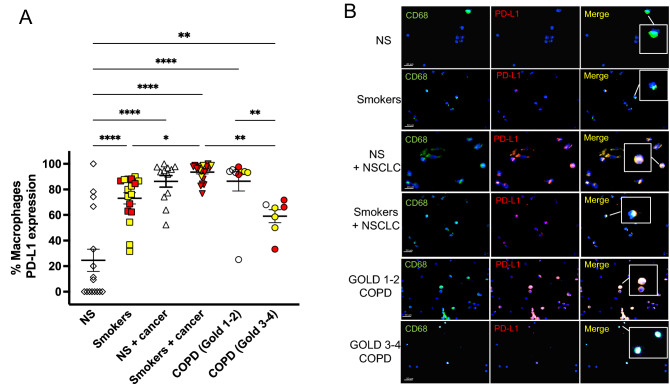
Similar PD-L1 expression in AMs from patients with GOLD 1–2 COPD and patients with NSCLC. Immunofluorescence analysis of PD-L1 expression by bronchoalveolar lavage (BAL) AMs from Cohort 2. (**A**) empty symbols represent never-smokers (NS); yellow symbols represent subjects who stopped smoking > 1 year prior to the study; red symbols represent current smokers at the time of the study. (**B**) representative immunofluorescence pictures showing CD68+ (green, as a marker of macrophages) and PD-L1+ (red) cells in bronchoalveolar lavage from all the cohort 2 study subjects. Nuclear DNA was labeled with DAPI (blue). Confocal microscopy was performed with 60X and 100X objectives. The insets show CD68 + alveolar macrophages (AMs) that were positive (NS + NSCLC, smokers + NSCLC, and GOLD 1–2 COPD) or negative (NS, smokers, GOLD 3–4 COPD) for PD-L1.

To extrapolate the effect of lung cancer (NSCLC) on PD-L1 pulmonary expression, we harvested alveolar macrophages (AMs) obtained by bronchoalveolar lavage from a second cohort of patients (Table 3). AMs from patients with GOLD 1–2 COPD and patients with NSCLC had the highest and similar PD-L1 expression vs. all the other subjects’ groups (Fig. 7A). Interestingly, PD-L1 expression was significantly higher in subjects with mild airflow limitation (GOLD 1–2) than those with severe airflow limitation (GOLD 3–4). A sub-analysis focused on the smoking status revealed that among all the ever smokers with NSCLC or COPD, there was no difference in PD-L1 expression between former (see yellow symbols) and current (see red symbols) smokers (Fig. 7A). Figure 7B shows representative pictures of the PD-L1 expression by BAL AMs from all groups.

### In vitro* studies*

#### PD-L1 mRNA expression levels in THP-1 cells after CSE exposure

To understand the dose and timing of CSE exposure, we used THP-1 macrophage cell lines. Treating THP-1 cells with 10% CSE significantly upregulated PD-L1 mRNA expression with a peak reached after 24 h (Supplemental Fig. S4). After this time point, or with CSE concentration higher than 10%, there was no significant change of PD-L1 mRNA expression.

#### PDL1 mRNA expression in BAL AMs in response to CSE exposure

**Figure 8:**
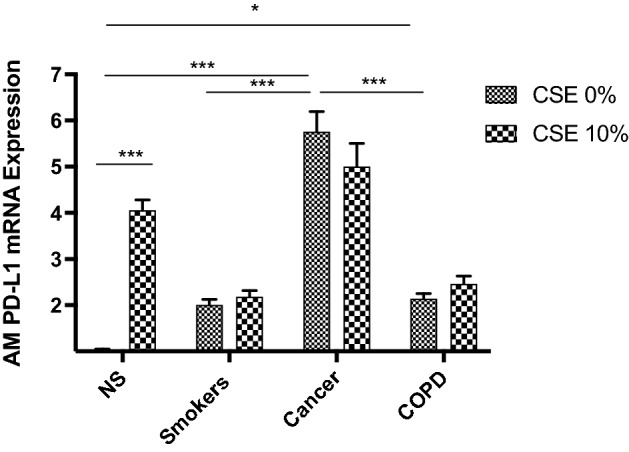
Analysis of PD-L1 mRNA expression levels in AMs from human BAL before and after 10% CSE at 24 h. AMs from BAL all groups were treated with CSE at 10% for 24 h and the expression of PD-L1 mRNA was assessed by real-time RT-PCR. PD-L1 mRNA expression is shown as fold change with respect to 0% CSE. Results were shown as means ± SEM. The statistical tests used in these analyses were one-way analysis of variance followed by Bonferroni's post-test. *P < 0.05, **P < 0.01, ***P < 0.001, ****P < 0.0001. NS = Never smokers without cancer or COPD. CSE = cigarette smoke extract.

The effect of CSE stimulation in vitro on PD-L1 mRNA expression in BAL AMs is shown in Fig. 8. Following 10% CSE stimulation for 24 h, we observed a significant increase in PD-L1 mRNA expression in the NS AMs only. In contrast, the CSE stimulation of AMs from other groups did not affect PD-L1 mRNA expression. This suggests that acute CS per se is sufficient to induce PD-L1 expression by AMs from individuals without pre-existing lung diseases or without prior smoke exposure. However, acute CS stimulation is unable to further induce PD-L1 expression by AMs from individuals who has been chronically exposed to CS or with underlying COPD to NSCLC.

## Discussion

For the first time, the present study characterizes by using cutting-edge digital spatial proteomic and genome-wide transcriptomic lung profiling combined with conventional immunofluorescence, the expression of the immune checkpoint PD-L1 in structural and inflammatory cells from two independent cohorts of COPD and lung cancer (NSCLC) patients and never- and ever-smoker controls.

First, we show that patients with milder stages of COPD express the highest levels of PD-L1 in alveoli, bronchioles, and vessels compared to patients with more severe COPD stages and ever- and never-smoker controls. We identified the bronchioles, where most NSCLCs occurs, as immune hot spots. In fact, in bronchioles only (and not in the other ROIs), the number of functionally active AMs was increased in milder COPD patients vs. other groups. Also, among all the COPD patients, PD-L1 expression, in particular in bronchioles, was significantly directly correlated with lung function and with the levels of functionally active AMs.

The incidence density of lung cancer is high in outpatients with established clinical COPD and occurs more frequently in patients with milder (GOLD 1–2) COPD^15^. The immune system is an essential barrier to tumor development^36^. The best evidence of the importance of immunosurveillance of tumors is inferred from the relationship between the immunosuppression used in transplanted patients and the higher incidence of cancers in this population. In milder stages of COPD, PD-L1 increases might prevent CS-induced sustained lung injury leading, in turn, to an exhausted/blunted immune phenotype more permissive to lung cancer. On the other hand, in more severe stages of COPD, an excessively active, nontolerant immune system would be a barrier to the development and progression of cancers. Still, it would lead to lung destruction known to occur in severe COPD, particularly emphysema^37^. It is essential to point out that, in some population-based cohorts, a higher incidence of NSCLC was associated with a higher degree of airway obstruction. However, none of the studies had the incidence of lung cancer as a primary outcome, and thus, many clinical parameters were missing^38,39^.

In the second cohort of patients, the highest AM PD-L1 expression levels were detected in patients with GOLD 1–2 COPD and patients with NSCLC, whom both had very similar levels of AM PD-L1 expression. Importantly, in this second cohort, we confirmed that patients with GOLD 1–2 COPD had significantly higher PD-L1 pulmonary levels than GOLD 3–4 COPD patients. The immune responses triggered by CS and characterizing COPD include the release of inflammatory cytokines, chemokines, and proteinases by innate immune cells such as AMs, leading to chronic pulmonary inflammation and structural changes in the lung^40^. Furthermore, the pulmonary inflammation often remains, even after CS cessation, suggesting a loss of immune modulation due to chronic antigenic stimulation. Therefore, it has recently been hypothesized that the COPD pathogenesis may be associated with a shift from the nonspecific innate response toward an adaptive immune response with hallmarks typical of autoimmune processes^10,11,41–43^. Chronic inflammation creates a favorable immunosuppressive microenvironment for tumor progression^44^. Previous studies reported dysregulation of the PD1/PD-L1 pathway in COPD, with increased PD-1 expression by T-cells from COPD patients compared to healthy NS^45^ and lower PD-L1 and/or PD-L2 expression in severe COPD AMs compared to NS^46^. In line with these data, we confirm that, while functionally active AMs from mild (GOLD 1–2) COPD patients had high PD-L1 expression levels, more severe (GOLD 3–4) COPD was associated with a reduced AM PD-L1 expression.

It is important to mention that the exuberant—yet aberrant—immune response observed in COPD patients is not dependent on immune checkpoints alone. In fact, other mechanisms occur in the COPD lung that might underlie the excessive immune activation in these patients despite the presence of high immune checkpoint levels. These mechanisms include off-targeted antigen presentation and regulatory mechanisms that facilitate the activation of extensive immune responses in the COPD lung. In line with these findings, the network of associations between the top proteins and genes in each tissue type revealed that CD68 and PD-L1 expression were associated with downregulation of oncosuppressive genes and genes involved in T cell responses, and upregulation of genes involved in cancer progression, immunosuppression, and chemoresistance.

Indeed, there are several mechanisms by which AMs contribute to COPD pathogenesis, such as a decreased AM phagocytic capacity^47,48^ and a CS-induced differentiation of monocyte alveolar precursors to M2 macrophages, which, by releasing metalloproteases, contribute to disease progression and severity^49^. The pathway analysis performed in our patient population confirmed that the PD-L1 protein-associated genes were involved in several macrophage responses including switching between M1 and M2 macrophages which, in turn, where associate with T cell activation. Thus, our and others’ data suggest that an increased checkpoint expression by AMs in COPD could, at least in part, contribute to the inability of AMs to negatively modulate the aberrant T-cell response observed in COPD^3,50^. Of note, the strong direct correlation we found between PD-L1 levels and numbers of functionally active (CD68^+^) AMs^51^ within the COPD patients hints at the active involvement of the AMs into the modulation of the adaptive immune responses in this patients’ group. Importantly, COPD is associated with better survival in advanced-stage lung cancer patients treated with immune checkpoint inhibitors^52^, especially in subjects with high plasma levels of IL-8 and IL-2R, both increased in activated macrophages. Accordingly, our novel data indicate higher levels of PD-L1^+^ AMs in milder COPD, suggesting that the AM-derived PD-L1, abundant in the earlier stages of COPD, may play a crucial role in NSCLC and represent a potential therapeutic target to prevent NSCLC onset and progression.

Nonetheless, it is still unclear whether macrophages are important in possible survival to lung cancer, as several trials until now did not show such a role. Thus, possible prediction of the role of macrophages with respect to lung cancer should be made carefully.

The PD-L1 levels in AMs from never smokers were the lowest across all the subjects, including smokers without COPD or NSCLC. However, no difference in PD-L1 expression was found in the AMs from current and former smokers. To validate the association between PD-L1 expression and smoking status, we assessed PD-L1 mRNA levels in AMs from BAL obtained from never-smokers, and smokers with NSCLC or COPD or none of the two diseases, before and after CS exposure. Interestingly, acute CS exposure did not affect PD-L1 expression levels in AMs from smokers, whether they had NSCLC or COPD. Still, it increased PD-L1 expression in AMs from never-smokers by over threefold. This observation suggests that acute exposure to CS per se can upregulate PD-L1 expression, but pre-existing chronic CS exposure (as in the ever-smoker controls) and/or the presence of COPD and lung cancer overwhelm the effect of acute CS exposure in inducing PD-L1 expression. This is in line with Wang et al., who documented a higher expression of PD-L1 by lung epithelial cells from smokers vs. non-smokers with lung cancer, and the ability of benzo(a)pyrene (BaP), the leading tobacco carcinogen, to induce PD-L1 expression on a lung epithelial cells line^53^.

Of note, we use for the first time Digital Spatial Profiling to study patients with COPD with and without cancer. The spatial profiling technology is the first available technique that allows the characterization of up to 100 proteins of interest and the whole transcriptome atlas in consecutive archived tissue sections, thus providing a comprehensive assessment of the transcriptomic and proteomic profile of the tissue in fewer steps than the conventional immunohistochemistry paired with methods such as laser capture microdissection.

One main limitation of this study is the lack of insight into how CS affects PD-L1 expression by AMs. Nonetheless, although mainly observational, our findings show for the first time a similar immunological background between mild stages of COPD and lung cancer, paving the way for future studies aimed at exploring the immune etiology underlying COPD and NSCLC onset and progression. Second, our sample size for the proteomic analyses is limited. However, the spatial profiling analyses generate sophisticated multiplexed information on cells and proteins of interest within multiple ROIs, which enhances the reproducibility of the data. Third, the main source of lungs in Cohort 1 was lung cancer surgery. In order to minimize the confounding effect of cancer, we selected patients with solitary pulmonary nodules (e.g., hamartomas) and the tissue taken was at least 10 cm away from the primary lesion. Although there is always the chance that cancer, even if benign and limited to a nodule, might have induced a systemic change in the immune microenvironment, GOLD 1–2 COPD patients still had a dramatic increase in PD-L1 expression compared to the other groups where the presence of lung cancer was similar. Last, in Cohort 2, we lack a group of subjects with both COPD and NSCLC. Future studies will need to investigate the immunological profile of patients having both diseases simultaneously, as often occurs in the clinical endeavor.

Altogether, our data point at a similar checkpoint inhibitor profile in lung cancer and milder stages of COPD. These observations represent a new resource in understanding the innate immune mechanisms underlying the link between COPD and lung cancer onset and pave the way to future studies focused on larger cohorts aimed at dissect the exact role of PD-L1 in the onset and progression of COPD in the context of lung cancer and vice-versa, and the mechanisms by which CS promotes tumorigenesis and COPD.

## Supplementary Information

Supplementary Information.

## Data Availability

Drs. Polverino and D’Agostino keep the raw data and figures for each of the experiments performed. The data are available upon request.
