# Supplementary material for: Similar programmed death ligand 1 (PD-L1) expression profile in patients with mild COPD and lung cancer
Source: Sci Rep. 2022 Dec 27;12:22402. doi: 10.1038/s41598-022-26650-9 (PMC9792927; doi:10.1038/s41598-022-26650-9)

Online Supplement

Similar Programmed Death Ligand 1 (PD-L1) expression profile in patients with mild COPD and lung cancer

Supplemental Figure 1. Correlation between total spatial PD-L1 expression levels and post-FEV1 (% predicted, **A)** and and CD68 expression levels **(B)** across all the study subjects. Results were shown as means ± SEM. The statistical test used in these analyses was Pearson correlation considering a 95% confidence interval.

Supplemental Figure 2. Correlation between total spatial PD-L1 expression levels and post-FEV1 (% predicted) in vessels **(A)** and alveoli **(B)** across all the study subjects. In **C** and **D**, the correlation between spatial PD-L1 expression and post-FEV1 (% predicted) in bronchioles across COPD patients and controls, respectively. Results are shown as means ± SEM. The statistical tests used in these analyses was Spearman correlation test considering 95% confidence interval.

Supplemental Figure 3: **Network of associations between top protein and genes and pathway analysis**: In **A** and **B**, vessel- and bronchiole-specific RNA-protein association network (restricted to top 10 RNAs per protein, ranked by p-value). Red nodes=Up-regulated proteins in GOLD 1-2 compared to GOLD 3-4; Blue nodes= Associated RNAs; Red edges = positive association between the RNA and the protein; Blue edges = negative association between the RNA and the protein; Edge widths are proportional to the association effect sizes. In **C**, the top 30 (ranked by p-value) C7 (immunologic signature gene sets) macrophage-related pathways enriched in PD-L1 associated genes. The vertical dotted line indicates a FDR=0.05.

Supplemental Figure 4. PD-L1 mRNA expression in THP-1 cells after CSE exposure. THP-1 cells were treated with CSE at 10% for the indicated time, and the expression of PD-L1 was assessed by real-time PCR. PD-L1 mRNA expression is shown as fold change with respect to 0% CSE.

Supplemental Figure 1

A

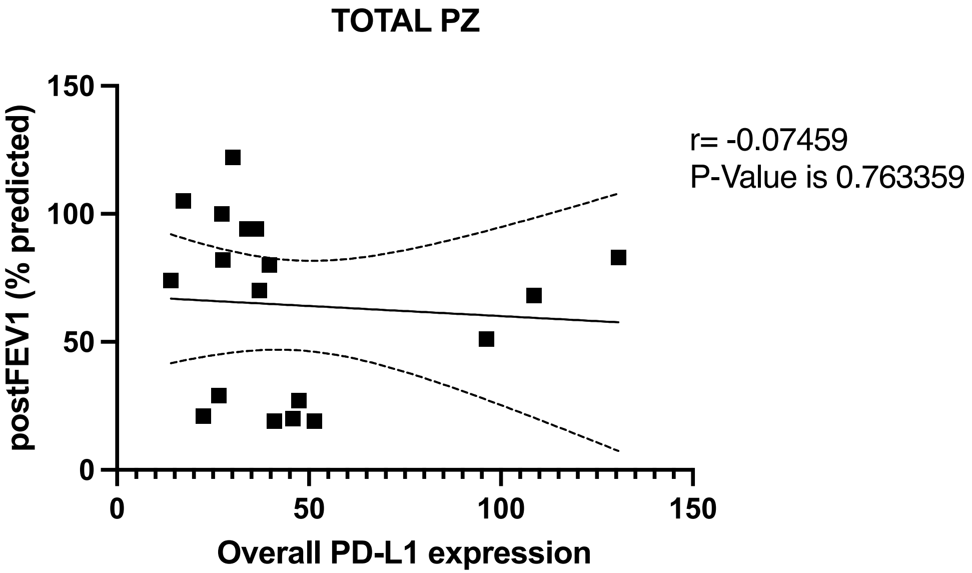

B

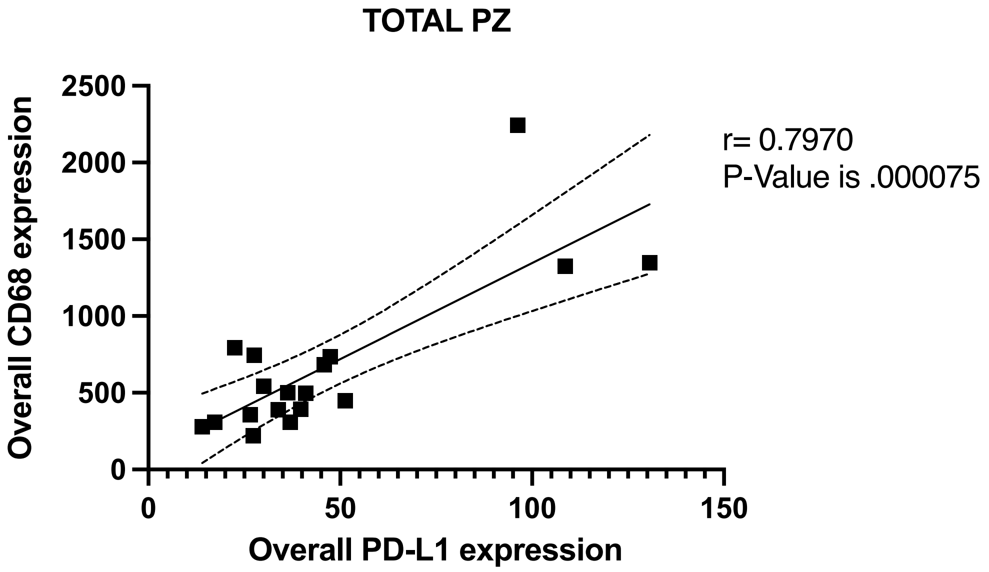

Supplemental Figure 2

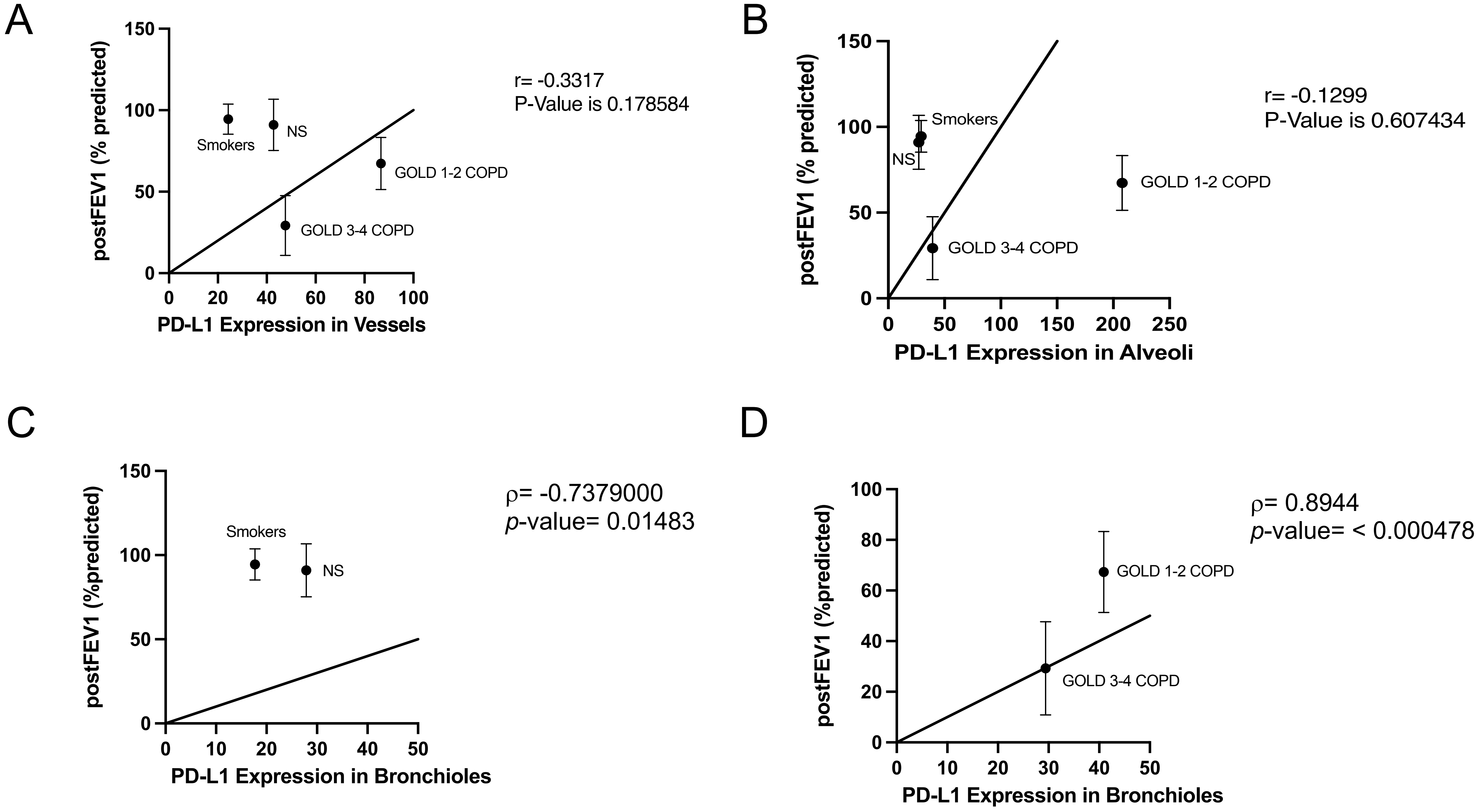

Supplemental Figure 3

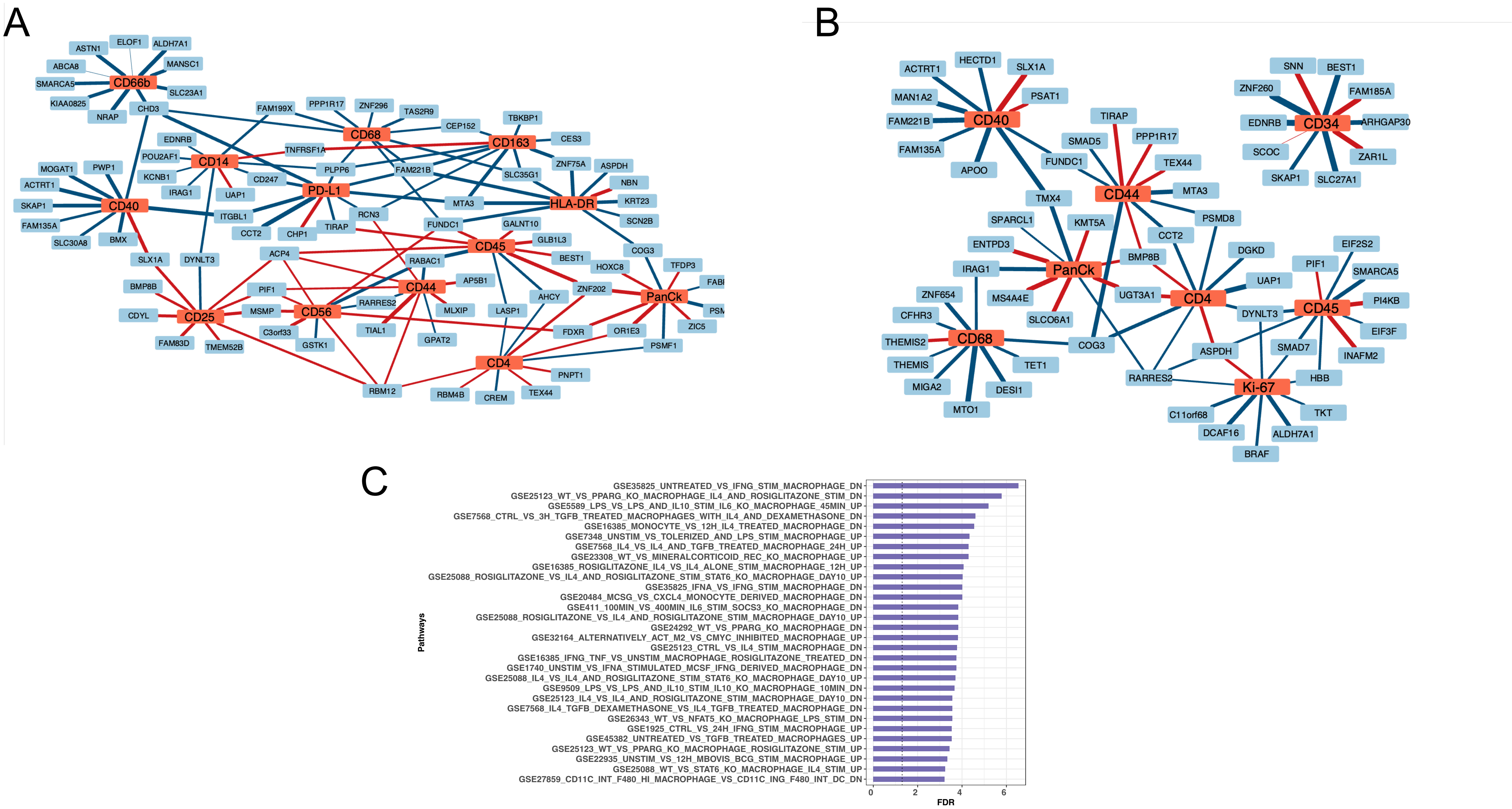

Supplemental Figure 4

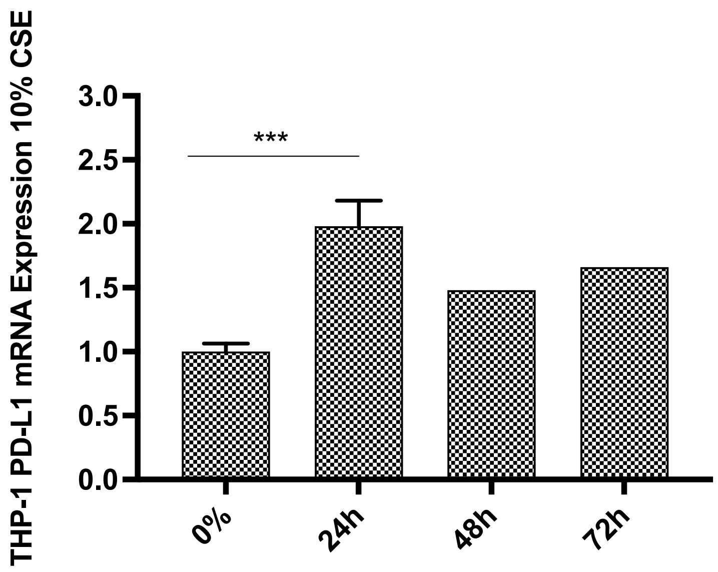

Supplement: Supplementary file 1 — Supplementary Information. [file 41598_2022_26650_MOESM1_ESM.pdf]
